# Exploratory study of the prevalence of food addiction and its relationship with executive functioning, depression, and reinforcement sensitivity in a sample of Mexican adults

**DOI:** 10.3389/fpubh.2023.1277681

**Published:** 2023-12-01

**Authors:** Marybeth Alejandra Téllez-Rodríguez, Adriana Amaya-Hernández, Mayaro Ortega-Luyando, Carlos Alberto Serrano-Juárez, Guillermina Yáñez-Téllez, Verónica Elsa López-Alonso, Juan Manuel Mancilla-Diaz, Rodrigo Erick Escartín-Pérez

**Affiliations:** ^1^Programa de Maestría en Psicología, División de Investigación y Posgrado, Facultad de Estudios Superiores Iztacala, Residencia en Neuropsicología, Universidad Nacional Autónoma de México, Tlalnepantla, Mexico; ^2^División de Investigación y Posgrado, Facultad de Estudios Superiores Iztacala, Nutrition Research Group, Universidad Nacional Autónoma de México, Tlalnepantla, Mexico; ^3^División de Investigación y Posgrado, Laboratory of Neurobiology of Eating, Facultad de Estudios Superiores Iztacala, Universidad Nacional Autónoma de México, Tlalnepantla, Mexico

**Keywords:** food addiction, executive function, depression, binge eating, reward sensitivity, obesity, body mass index

## Abstract

**Introduction:**

The study of food addiction (FA) has become relevant due to its high prevalence, the negative impact on quality of life, and its association with neuropsychological and psychiatric symptoms. Several studies have provided scientific support for these associations, however, the results are contradictory. Additionally, studies have unsuccessfully elucidated the true nature of the failures in executive functioning in people with FA symptomatology, particularly when it comes to executive deficits. Therefore, the purpose of this research was to establish whether the presence of executive dysfunction, depressive symptoms and binge eating problems, as well as high reward sensitivity entails a greater severity in FA traits and high body mass index (BMI) in a sample of Mexican adults.

**Methods:**

The sample consisted of Mexican men and women between 21–59 years (*n* = 36); who completed self-report questionnaires and performance tests to measure the study variables. Additionally, BMI was estimated with self-reported height and weight.

**Results:**

Our results showed that a high number of FA symptoms were associated with higher executive dysfunction scores, greater reward sensitivity, and more severe depressive and binge eating problems. Furthermore, factors that are more strongly associated with higher scores of FA include severe executive deficits, greater activation of the punishment avoidance system, and persistence in the search for reward when the depressive symptoms increased. The factors that best explained changes in the estimated BMI of women were a decreased crystallized intellectual capacity and the inability to control food intake as the number of FA symptoms increased.

**Discussion:**

In summary, the cognitive functioning profile characterized by general failure of the executive functioning, as well as a greater activation of the Punishment Avoidance System and persistence in the search for reward, were associated with greater severity of FA symptoms, especially when the depressive symptomatology was severe. In parallel, the psychopathology in participants associated with FA confirms the contribution of anxious and depressive symptomatology and borderline personality traits which could facilitate the expression of clinically relevant FA symptoms in women. Finally, we found that decreased crystallized intellectual capacity and inability to control food intake were linked to higher BMI when the number of FA symptoms increased.

## 1 Introduction

Although the DSM-5 TR (1) and the ICD-11 (2) do not formally recognize food addiction (FA) as a psychiatric disorder, the complexity of this condition and the evidence that FA has a biological basis comparable to substance addiction (with altered reward circuitry functioning) has generated scientific debate in recent years (3, 4). Some researchers propose that FA is an addictive process in which behaviors and cognitions are present and produce non-adaptive behavioral patterns similar to those observed in patients with substance addictions (5). However, several reports agree that there is no scientific evidence to categorize any micronutrient, food component, or standard food additive as addictive (6). Consequently, FA has been considered a behavioral addiction (7), although the clinical manifestations are still more comparable to substance use disorders with hyper-palatable food as the object of desire.

FA is defined as excessive and uncontrollable food consumption (especially, but not exclusively, hypercaloric foods high in carbohydrates and fats) associated with significant impairment in multiple domains of personal functioning (8). Similar to substance addiction, when FA symptoms are severe, altered salience, mood changes, tolerance, withdrawal, consumption of highly palatable and processed foods despite knowing their negative consequences, and relapse are observed (9, 10). There is evidence that in both animal models and humans with symptoms of FA, palatable foods can result in abnormal brain activation in areas of the amygdala, frontal cortex, and reward circuit (4, 11, 12), D2 receptor downregulation (13–15), and cortical dysfunction that interferes with appropriate behavioral regulation, primarily characterized by impulsivity (11, 16).

FA has a high comorbidity with eating pathology, in which excessive food consumption is present. Indeed, a significant number of individuals with obesity may present FA symptoms (17), and a high percentage of patients (55–57%) with binge eating comorbidly present FA symptoms (18, 19). Moreover, FA is more frequent in women, reaching a 2:1 ratio compared to men (20).

Traits of FA are simultaneously present in patients with anxious and depressive symptoms, and impaired executive functioning, which has made it difficult to fully characterize FA as a well-defined psychiatric entity (21–23). Although some researchers failed to find alterations in executive functioning (attention, cognitive flexibility, and inhibition) in individuals with FA (17), other groups have reported that the severity and number of FA symptoms correlate with deficits in decision-making and attentional processes (24), as well as in inhibition and cognitive flexibility (22). While it is not entirely clear which cognitive domains are most affected in individuals with FA, the behavioral pattern of these persons is certainly a condition that contributes significantly to excessive weight gain and body fat accumulation, although not all patients with obesity necessarily suffer from FA (3).

In this context, the high prevalence of obesity and overweight in adolescent and adult populations worldwide has led to the need for further research into the factors that contribute to the increase in body weight, which impairs health and quality of life. Therefore, the study of FA may be considered an alternative to better understanding the problem of body fat accumulation associated with abnormal eating patterns. Accordingly, the present study aimed to explore whether altered executive functioning, depressive symptoms, binge eating problems, and high reward sensitivity explain the increased severity of FA traits and BMI in a sample of Mexican adults.

## 2 Methods

### 2.1 Participants

The sample was recruited using an accidental non-probabilistic procedure through advertisements displayed on social media websites (Facebook) and on the official website of a public university. The final sample in this study consisted of 36 women and men with Mexican nationality and residence aged between 21 and 59 years (*M* = 35.8, *SD* = 11.72, 63.9% women). Individuals who did not complete the self-report questionnaires, neuropsychological tests, or online interviews were excluded from the study. Similarly, those who scored less than one standard deviation (SD) below the mean on the Shipley-2 (<85) or who scored above the cutoff on the psychopathology subscales of the Personality Assessment Inventory were not included in the final sample.

### 2.2 Evaluation instruments

#### 2.2.1 Yale food addiction scale

We used the validated version of the YFAS for Mexican adults to assess FA traits. This self-reported questionnaire consists of 25 items with frequency response options (never, once a month, 2–4 times a month, 2–3 times a week, four or more times a week, or daily), which allows to determine whether someone may have FA, as well as, dichotomous response options (yes/no), which assess severe problems with food consumption. The presence of FA was determined if a person exhibited at least three positive criteria for FA and clinically significant impairment (positive responses to items 15 and 16). This version of YFAS has an internal consistency of α = 0.79 in the Mexican population (25).

#### 2.2.2 Body mass index

We calculated BMI with self-reported weight and height and categorized according to the World Health Organization's cut-points (26) for normal weight (18.5–24.9), overweight (25.0–29.9), or obesity (≥30).

#### 2.2.3 Shipley-2

The Shipley-2 Brief Intelligence Scale was used to assess intellectual ability and cognitive functioning using verbal and non-verbal subtests. This scale has a mean of 100 and a SD of 15, so scores below 85 indicate probable cognitive impairment. The Shipley-2 scale is a brief but reliable measure of cognitive function and impairment. It has an internal consistency of α = 0.80, and scores between 85 and 115 are considered statistically normal for the Mexican population (27).

#### 2.2.4 Dysexecutive questionnaire (DEX-Sp)

This self-report instrument allows the assessment of general dysexecutive symptoms in adults from an ecological perspective. It consists of 20 items with frequency response options (never, sometimes, often, often, very often, almost always) and has an internal consistency of α = 0.91 in the Mexican population (28).

#### 2.2.5 Iowa gambling task (computerized version of PsyToolkit)

This test assesses decision-making ability. In the test, the participant must choose between four buttons (corresponding to the 100 betting opportunities, instead of the cards in the original version) and receives feedback indicating a gain or loss of money. Two buttons (A and B) are not beneficial as the final loss of money is greater than the overall gain. Buttons C and D, on the other hand, are advantageous because they provide small final gains. The participants were informed that the goal of the task was to accumulate as much money as possible. IGT performance was determined by subtracting the total number of disadvantageous options from the total number of advantageous options and expressed as a direct score (total net score) (29, 30).

#### 2.2.6 Go/no-go task (computerized version of PsyToolkit)

This test was used to assess inhibitory control. The text “Go” or “No-go” was presented on the screen, with the first option requiring the user to press a key within 2 seconds, while the second option required the user to avoid pressing the key for 2 seconds. The text “Go” was presented 202 times, while stimuli containing “No-go” appeared randomly 48 times. The number of commission errors made (pressing the key with the text No-go) was used to measure performance in this task, and we expressed it as a direct score. The higher the number of commission errors, the lower the inhibitory control (29, 30).

#### 2.2.7 Wisconsin card sorting inspired task (computerized version of PsyToolkit)

This task is designed to assess cognitive flexibility. It involves matching cards based on various criteria such as color, number, or objects form on the card. After each response, participants received feedback indicating whether they should continue using the same strategy or switch to a different one for card pairing. Participants established a category by correctly matching ten cards in a row, after which the rule changed. The evaluation continued until participants completed six categories or reached 60 trials. To quantify performance on this task, we quantified perseverative errors and expressed them as percentages, which reflects the tendency to persist in the previous criterion (29, 30).

#### 2.2.8 PAI personality assessment inventory (short version)

This instrument provides a comprehensive assessment of personality and psychopathology in adults over 18 years. The short version consists of 165 items scored on a 4-point Likert-type scale (strongly disagree-strongly agree). The items are grouped into 22 major scales: (a) 4 Validity Scales (Inconsistency, Infrequency, Negative Impression, and Positive Impression), (b) 11 Clinical Scales (Somatic Complaints, Anxiety, Anxiety-related Disorders, Depression, Mania, Paranoia, Schizophrenia, Borderline Traits, Antisocial Traits, Alcohol Problems, and Drug Problems), (c) 5 Treatment Consideration Scales (Aggression, Suicidal Ideation, Stress, Lack of Social Support, and Treatment Rejection), and (d) 2 Interpersonal Relationship Scales (Dominance and Warmth). The inventory yields t-scores, higher scores indicate a higher level of the variable under study. This instrument has an internal consistency of α = 0.74 in neurotypical samples and α = 0.81 in clinical samples (31).

#### 2.2.9 Beck depression inventory-II

This instrument allows the detection and assessment of the severity of depressive symptoms. It is a self-report instrument consisting of 21 items with a Likert scale ranging from 0 to 3 and a total score ranging from 0 to 63. In the Mexican population, the cut-off points are 0–13, minimally depressed; 14–19, mildly depressed; 20–28, moderately depressed; and 29–63, severely depressed. The BDI-II has an internal consistency of α = 0.87 in the Mexican population (32).

#### 2.2.10 Binge eating scale

BES assesses the cognitive-behavioral traits of binge eating. It is a self-report instrument composed of 16 items, 8 evaluate behavioral aspects, and the other half assess cognitions and feelings associated with binge eating. Each item includes four statements that measure the severity of the trait on a scale from 0 to 3. In the Mexican population, the cut-off point for the presence of binge eating problems is 17 (33). In addition, the severity of binge eating problems was quantified using the following scores: <17 points do not suffer from binge eating problems, scores between 18 and 26 points indicate the presence of moderate binge eating problems, and scores >27 indicate the presence of the syndrome to a severe degree (34). Validation of the BES in the Mexican population showed internal consistency of α = 0.91 and α = 0.90 for the feelings and cognitions, and behavioral manifestations subscales, respectively (33).

#### 2.2.11 Reinforcement sensitivity theory of personality questionnaire

This scale measures reward sensitivity. It consists of 79 statements that assess the anxiety-related Fight-Flight-Freeze System (FFFS) and the anxiety-related Behavioral Inhibition System (BIS), as well as four factors of the Behavioral Approach System (BAS), including Reward Interest, Goal Persistence, Reward Reactivity, and Impulsivity. These statements are scored on a Likert scale from 0 to 3 (0 = not at all, and 3 = very much) with a ranging score of 0–237 points. The higher scores indicate greater sensitivity to reward (35). In the Mexican population, the RST-PQ showed an internal consistency of α = 0.89 (36).

### 2.3 Procedure

Participants who responded to the published announcement first completed a Google^®^ Form. In this Form, we explained in detail the purpose of the study, obtained informed consent, and collected general information from the participants (including height and weight). Shipley-2 and PAI were applied to assess the inclusion criteria for the study during a videoconference meeting (Zoom^®^).

Participants who met the inclusion criteria received the links to the Google^®^ Forms containing the self-report instruments with two different order sequences: (YFAS, DEX-Sp, BES, BDI-II, RST-PQ) and (RST-PQ, BDI-II, BES, DEX-Sp, YFAS). Moreover a new videoconference session was scheduled to administer the computerized neuropsychological tests (IGT, Go/No-go Task, and WCST) to assess executive functioning while participants shared their screen. This final session lasted an average of 45 min, and the participants were instructed to be free of distractions during this time.

In exchange for taking part in the study, participants received a detailed report of the evaluations conducted and, when requested, were referred to the psychological care center of a public university. All assessments and interviews were conducted online due to the restriction of in-person activities established by health authorities during the COVID-19 pandemic. Participants were evaluated between May and December 2022.

### 2.4 Statistical analyses

Data analysis was conducted using IBM SPSS Statistics^®^ version 25.0 and JASP^®^ version 0.14.1. We employed *X*^2^ tests (with Yates' continuity correction) and *t-*tests to assess the relationship between food addiction and participants' gender and BMI. Additionally, we utilized ANOVAs and *t-*tests to compare variables between participants with and without FA. When the assumption of homogeneity of variances was violated (Levene's test), we applied the Welch or Brown-Forsythe correction method, as appropriate. Following significant ANOVAs, we performed Bonferroni *post hoc* tests for multiple comparisons. To examine linear associations between variables while controlling for sex, BMI, and the presence of FA, we calculated partial correlation coefficients (*rho*). Finally, we employed multiple linear regression (MLR) to determine the impact of different variables on changes in FA scores and estimated BMI in women. We assessed the goodness of fit of the regression models using the corrected r-squared (*r*${\;}_{c}^{2}$) and ensured that the variance inflation factor (*VIF*) was <3 to avoid collinearity in the models. All hypothesis tests were conducted with an α level of 0.05 to determine statistical significance. Effect sizes were calculated using Cohen's *d* or η^2^, as appropriate.

### 2.5 Ethics

The study was conducted following the Declaration of Helsinki, and the Ethics Committee of FES Iztacala UNAM approved the evaluation protocol (CE/FESI/052022/1516). All subjects were informed about the study aims, and all provided written informed consent.

## 3 Results

### 3.1 Sample characterization and FA prevalence

The final sample consisted of 36 participants who met the inclusion criteria of the study and signed the informed consent Form. They were predominantly women, living in the metropolitan area of Mexico City, between 21–59 years (*M* = 35.8, *SD* = 11.72), with undergraduate or postgraduate or high school education (Table 1).

**Table 1:** Sociodemographic characteristics.

	***n* (%)**
**Sex**	
Females	23 (63.9)
Males	13 (36.1)
**Residence**	
Mexico City	13 (36.1)
State of Mexico	12 (33.3)
Other states	11 (30.6)
Age^*^	35.8^*^ (11.72)
**Education**	
High school	8 (22.2)
Undergraduate	18 (50.0)
Graduate	10 (27.8)

Values are expressed as frequencies and percentages (%) of the total sample (*n* = 36) or as means (X) and their standard deviations (SD).

* Data expressed as means and SD.

Intellectual functioning scores on the Shipley-2 subscales showed that 11.1% to 13.9% of the participants scored below average without indicating cognitive impairment, as no one scored less than one SD below the mean. In addition, 66.7% of the participants had mild to severe deficits in executive functioning (DEX-Sp), and 27.8% of the sample had moderate to severe depressive symptoms on the BDI-II. Similarly, 44.4% of the participants had moderate to severe scores on the Binge Eating Scale. Eighty-six percent of the participants had an estimated BMI corresponding to overweight or obesity. Finally, 22.2% of the participants met the YFAS criteria for the presence of FA (3 positive symptoms + clinically significant impairment) (Table 2).

**Table 2:** Neuropsychological characteristics, psychopathology, BMI and FA.

	***n* (%)**
**Vocabulary (Shipley-2)**	
Expected average or above	31 (86.1)
Below average within 1 SD	5 (13.9)
**Abstraction (Shipley-2)**	
Expected average or above	32 (88.9)
Below average within 1 SD	4 (11.1)
**Cognitive ability (Shipley-2)**	
Expected average or above	32 (88.9)
Below average within 1 SD	4 (11.1)
**Dysexecutive symptoms (DEX-Sp)**	
Average or above	12 (33.3)
Below average or mild failures	8 (22.2)
Moderate or severe failures	16 (44.5)
**Depressive symptomatology (BDI-II)**	
Minimal	23 (63.9)
Mild	3 (8.3)
Moderate	5 (13.9)
Severe	5 (13.9)
**Cognitive-behavioral symptoms of binge eating (BES)**	
Absence of binge eating	20 (55.6)
Moderate binge eating	12 (33.3)
Severe binge eating	4 (11.1)
**Estimated BMI** ^*^	30.63^*^ (7.18)
Normal weight (18.5–24.9)	5 (13.9)
Overweight (25.0–29.9)	16 (44.4)
Obesity (≥30)	15 (41.7)
**Food addiction (YFAS)**	
Absent	28 (77.8)
Present	8 (22.2)

Values are expressed as frequencies and percentages (%) of the total sample (*n* = 36) or as means (X) and their standard deviations (SD). DEX-Sp, Dysexecutive Questionnaire; BDI-II, Beck Depression Inventory-II; BES, Binge Eating Scale; BMI, Body Mass Index; YFAS, Yale Food Addiction Scale.

* Data expressed as means and SD.

Moreover, we observed that the proportion of participants fulfilling the criterion for the presence of FA was higher in females, as 26.1% of the 23 women fulfilled the criterion compared to 15.4% of men. However, this difference did not reach statistical significance (*X*^2^ = 0.11, *p* > 0.05, Yates continuity correction). When we compared the proportion of participants meeting the FA criterion concerning their estimated BMI, we found that in the total sample and in men, those with a BMI > 30 were more frequently meeting the YFAS criterion (*X*^2^ = 8.98, *X*^2^ = 7.88, *p* > 0.05). A similar trend was observed in women, although it was not statistically significant (Table 3).

**Table 3:** BMI and FA.

	**BMI < 25**	**25 ≤ BMI < 30**	**BMI ≥30**		
**Food addiction**	***n*** **(*****%*****)**	***n*** **(*****%*****)**	***n*** **(*****%*****)**	*X*^2^▸	***p*** **-value**
**Total (*****n** =* **36)**					
Absent (*n =* 28, 77.8%)	5 (13.89)	15 (41.67)	8 (22.22)	**8.98**	**0.011**
Present (*n =* 8, 22.2%)	0 (0)	1 (2.78)	7 (19.44)		
**Females (*****n** =* **23)**					
Absent (*n =* 17, 73.9%)	3 (13.04)	7 (30.43)	7 (30.43)	3.34	0.189
Present (*n =* 6, 26.1%)	0 (0)	1 (4.35)	5 (21.74)		
**Males (*****n** =* **13)**					
Absent (*n =* 11, 84.6%)	2 (15.38)	8 (61.54)	1 (7.69)	**7.88**	**0.019**
Present (*n =* 2, 15.4%)	0 (0)	0 (0)	2 (15.38)		

Values are expressed as frequencies and percentages (%) of the total sample. X^2^▸ = X^2^ with Yates continuity correction. BMI, Body Mass Index. Statistically significant data in bold text.

### 3.2 Neuropsychological characteristics and psychopathology

Estimated BMI, age, intellectual ability, as well as assessments of decision-making (IGT), cognitive flexibility (WCST), and Alcohol Problems and Drug Problems (PAI) in participants who met or did not meet the criterion for the presence of FA did not differ significantly. However, when compared dysexecutive symptoms, cognitive-behavioral binge eating problems, and reward sensitivity (total score RST-PQ) between participants with and without symptoms of FA we found that those who met YFAS criteria scored significantly higher [*t*_(34)_ = 2.3, *p* < 0.05; *t*_(23)_ = 3.98, *p* < 0.001; *t*_(23)_ = 2.89, *p* < 0.01; respectively] (Table 4).

**Table 4:** FA, BMI, and neuropsychological evaluations, total sample.

	**NFA (*n =* 28)**	**FA (*n =* 8)**	***t***	***p*-value**	***d***
**Measures**					
BMI°	29.75 (7.69)	33.69 (4.04)	1.39	0.175	0.64
Weight°	80.64 (22.21)	87.01 (13.09)	0.78	0.441	0.35
Height°	1.64 (0.084)	1.61 (0.084)	−1.14	0.262	0.36
Age	36.54 (11.89)	33.13 (11.43)	−0.72	0.476	0.29
**Intellectual ability**					
Vocabulary Shipley-2^▿^	109.86 (3.85)	107.13 (5.82)	−1.58	0.124	0.55
Abstraction Shipley-2^▿^	102.07 (8.53)	104.00 (9.04)	0.56	0.581	0.22
Total^▿^	109.75 (6.68)	112.13 (7.00)	0.88	0.386	0.35
**Decision-making (IGT)**					
Total net score	−0.71 (36.17)	9.50 (27.21)	0.74	0.466	0.32
**Cognitive flexibility (WCST)**					
Perseverative errors (%)	16.86 (8.21)	15.88 (9.43)	−0.29	0.774	0.11
Commission errors	3.79 (4.09)	3.38 (2.14)	−0.38^▸^	0.707	0.13
**Psychopathology (PAI)**					
Drug problems^□^	47.71 (7.02)	45.13 (1.55)	−1.81^▸^	0.080	0.51
Alcohol problems^□^	48.82 (9.69)	44.63 (1.77)	−1.21	0.236	0.60
**Dysexecutive symptoms (DEX-Sp)**					
Dysexecutive score	39.75 (9.69)	**50.63 (13.08)** ^*^	2.30	**0.028**	**0.95**
**Binge eating (BES)**					
Total score	14.04 (7.39)	**27.5 (11.67)** ^**^	3.98	**0.001**	**1.38**
**Depressive symptoms (BDI-II)**					
Total score	11.43 (8.60)	22.13 (15.59)	−1.86^▸^	0.099	0.85
**Reward sensitivity (RTS-PQ)**					
Total score	106.4 (31.54)	**142.6 (30.20)** ^**^	2.89	**0.007**	**1.17**

Values are expressed as means and their SDs. Standard scores have a mean of 100 and a SD of 15, while t-scores have a mean of 50 and a SD of 10. °Estimates based on questionnaire responses; ^▿^Standard score; ^□^t-score; d = effect size, Cohen's d. ^▸^Welch's homogeneity correction after significant test for equality of variances (Levene's). NFA, non-food addiction; FA, food addiction; BMI, body mass index; IGT, Iowa Gambling Test; PAI, Personality Assessment Inventory; DEX-Sp, Dysexecutive Questionnaire; BES, Binge Eating Scale; BDI-II, Beck Depression Inventory-II; RST-PQ, Reinforcement Sensitivity Theory of Personality Questionnaire.

* *p* < 0.05,

** *p* < 0.01 vs. NFA. Statistically significant data in bold text.

Estimated BMI, age, and assessments of decision-making (IGT), cognitive flexibility (WCST), and Alcohol Problems and Drug Problems (PAI) in females did not differ significantly by the effect of the presence of FA. However, when comparing intellectual ability in females with and without FA, we found that those with FA scored significantly lower [*t*_(21)_ = −2.22; *p* < 0.05] on the Vocabulary subscale of the Shipley-2. Furthermore, dysexecutive, binge eating, and reward sensitivity scores were significantly higher in females with FA [*t*_(21)_= 4.18, *p* < 0.01; *t*_(21)_ = 2.77, *p* < 0.05; *t*_(21)_ = 0.55, *p* < 0.05; respectively] (Table 5). Males with FA had a lower percentage of perseverative errors on the WCST and lower scores on Drug Problems (PAI) compared with those without FA [*t*_(11)_ = −3.88, *p* < 0.01, *t*_(11)_ = −2.59, *p* < 0.05, respectively] (Supplementary Table 1).

**Table 5:** FA, BMI, and neuropsychological evaluations in females.

	**NFA (*n =* 17)**	**FA (*n =* 6)**	***t***	***p*-value**	***d***
**Measures**					
BMI°	30.88 (8.72)	34.24 (4.61)	0.89	0.384	0.48
Weight°	79.03 (25.40)	84.3 (13.79)	0.48	0.637	0.26
Height°	1.59 (0.06)	1.57 (0.04)	−1.06	0.303	0.39
Age	42.12 (11.67)	33.17 (12.80)	−1.58	0.130	0.73
**Intellectual ability**					
Vocabulary Shipley-2^▿^	110.47 (4.16)	**105.67 (5.68)** ^*^	−2.22	**0.038**	**0.96**
Abstraction Shipley-2^▿^	102.59 (7.72)	103.67 (10.65)	−0.27	0.792	0.12
Total^▿^	108.71 (6.14)	110.67 (7.58)	−0.63	0.533	0.28
**Decision-making (IGT)**					
Total net score	−11.65 (33.75)	2.67 (28.54)	−0.93	0.366	0.46
**Cognitive flexibility (WCST)**					
Perseverative errors (%)	15.12 (6.68)	18.50 (9.57)	0.95	0.351	0.41
Commission errors	3.47 (3.76)	4.17 (1.72)	0.43	0.670	0.24
**Psychopathology (PAI)**					
Drug problems^□^	45.18 (2.40)	45.50 (1.64)	0.30	0.765	0.16
Alcohol problems^□^	46.47 (4.36)	44.83 (2.04)	−0.88	0.391	0.48
**Dysexecutive symptoms (DEX-Sp)**					
Dysexecutive score	35.82 (8.14)	**54.17 (12.12)** ^**^	4.18	**0.001**	**1.78**
**Binge eating (BES)**					
Total score	14.29 (7.10)	**29.50 (12.79)** ^*^	2.77^▸^	**0.032**	**1.47**
**Depressive symptoms (BDI-II)**					
Total score	11.18 (8.80)	11.18 (8.80)	2.28^▸^	0.062	1.21
**Reward sensitivity (RTS-PQ)**					
Total score	101.65 (34.04)	**143.00 (34.35)** ^*^	0.55	**0.019**	**1.21**

Values are expressed as means and their SDs. Standard scores have a mean of 100 and a SD of 15, while t scores have a mean of 50 and a SD of 10. °Estimates based on questionnaire responses; ^▿^Standard score; ^□^t score; d = effect size, Cohen's d. ^▸^ Welch's homogeneity correction after significant test for equality of variances (Levene's). NFA, non-food addiction; FA, food addiction; BMI, body mass index; IGT, Iowa Gambling Test; PAI, Personality Assessment Inventory; DEX-Sp, Dysexecutive Questionnaire; BES, Binge Eating Scale; BDI-II, Beck Depression Inventory-II; RST-PQ, Reinforcement Sensitivity Theory of Personality Questionnaire.

* *p* < 0.05,

** *p* < 0.01 vs. NFA. Statistically significant data in bold text.

**Figure 1:**
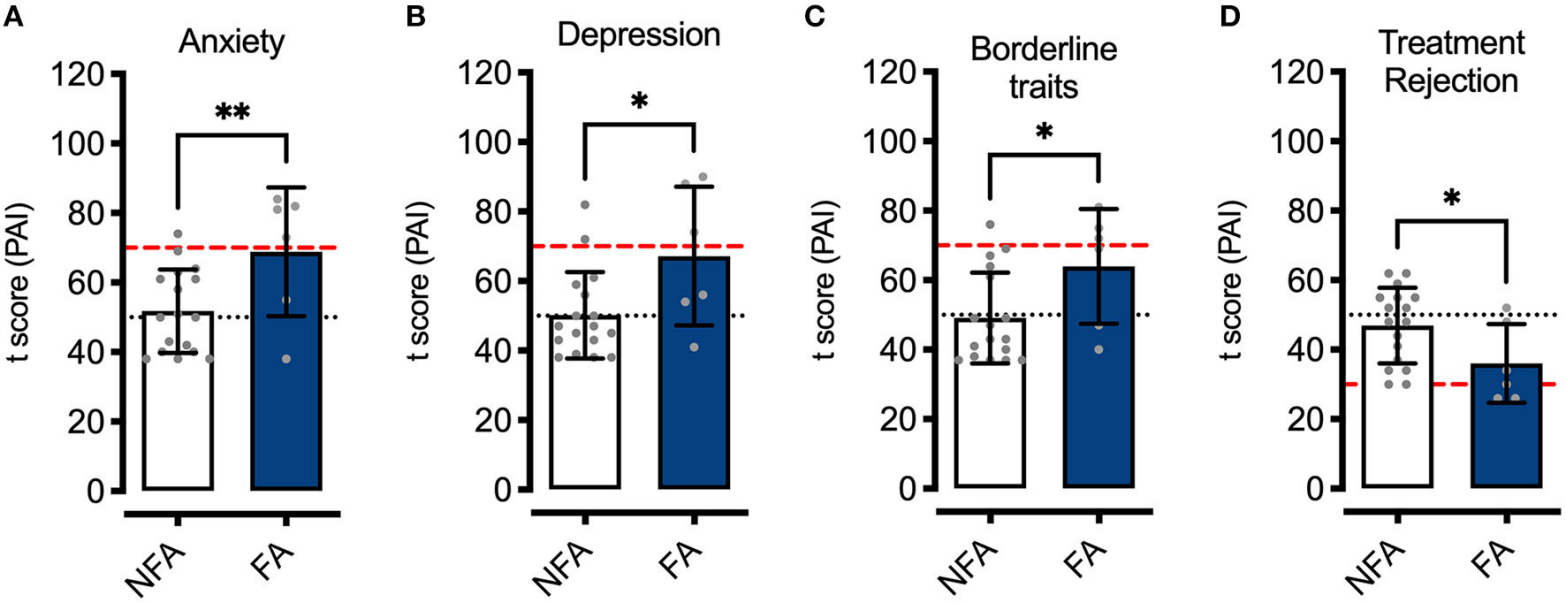
Anxiety, depression, borderline features, and treatment rejection (PAI). T-scores of females with FA (FA, *n* = 6) and without FA (NFA, *n* = 17) (3 symptoms + clinically significant impairment YFAS) on the Anxiety **(A)**, Depression **(B)**, Borderline traits **(C)**, and Treatment Rejection **(D)** scales. Data are expressed as means ± SD, and gray dots represent the individual scores of participants of each group. The black horizontal dotted lines show the mean (50) t-scores of the normative population, while the red dashed line represents the t-score at a distance of 2 SD from the mean. *p < 0.05, **p < 0.01.

When we compared the results of the application of the PAI in females with and without FA, we found that those who met the YFAS criteria scored significantly higher on the Anxiety, Depression, and Borderline Features scales [*t*_(21)_ = 2.6, *p* < 0.01; *t*_(21)_ = 2.46, *p* < 0.05; *t*_(21)_ = 2.24, *p* < 0.05; respectively], while on average they scored lower on the Treatment Rejection scale [*t*_(21)_ = 2.1, *p* < 0.05] (Figure 1). No statistically significant differences were found when comparing these variables among the males in the sample.

### 3.3 Differences in executive functioning, reward sensitivity, and binge eating problems of individuals with food addiction

When comparing YFAS subscale-specific scores as a function of BMI in the total sample and females, no significant differences were observed (Supplementary Table 2). In contrast, when we grouped the sample by the severity of dysexecutive symptoms, it was found that both the total sample and females had significantly higher scores on the subscales of the YFAS (Increased Consumption, Tolerance, Reduced Social Activities, and Distress Dysfunction) specifically when severe executive dysfunction was found [*F*_(2,33)_= 5. 6, *p* < 0.01; *F*_(2,33)_ = 5.50, *p* < 0.01; *F*_(2,33)_ = 5.72, *p* < 0.01; *F*_(2,33)_ = 4.79, *p* < 0.05, total sample; *F*_(2,20)_= 6.12, *p* < 0.01; *F*_(2,20)_ = 7. 88, *p* < 0.01; *F*_(2,20)_ = 5.41, *p* < 0.05; *F*_(2,20)_ = 8.26, *p* < 0.01, in females] and effect sizes were systematically larger in females (Table 6). Finally, there were no significant differences among YFAS subscales scores associated with the severity of executive dysfunction in males (Supplementary Table 3).

**Table 6:** YFAS subscales and dysexecutive symptoms (DEX-Sp).

	**No dysfunction**	**Mild-moderate dysfunction**	**Severe dysfunction**			
**Food addiction (YFAS)**	***X (SD)***	***X (SD)***	***X (SD)***	***F***	***p*** **-value**	*η^2^*
**Increased consumption**						
Total sample	13.86 (6.47)	**11.86 (5.22)** ^**+**^	**21.00 (8.25)** ^**^	5.60	**0.008**	**0.25**
Females	13.0 (6.36)	**12.33 (5.68)** ^**+**^	**24.13 (9.43)** ^**^	6.12	**0.008**	**0.38**
**Tolerance**						
Total sample	2.14 (2.48)	4.00 (2.51)	**5.57 (3.08)** ^**^	5.50	**0.009**	**0.25**
Females	2.11 (2.42)	5.17 (1.47)	**7.00 (3.25)** ^**^	7.88	**0.003**	**0.44**
**Continuous craving**						
Total sample	5.86 (1.79)	4.63 (1.30)	5.93 (1.33)	2.16	0.131	0.12
Females	5.44 (2.00)	4.66 (1.50)	6.63 (1.06)	2.68	0.093	0.21
**Reduced social activities**						
Total sample	1.21 (1.63)	1.63 (1.41)	**3.86 (2.88)** ^**^	5.72	**0.007**	**0.26**
Females	1.44 (1.81)	2.00 (1.41)	**5.00 (3.38)** ^*^	5.41^▸^	**0.016**	**0.34**
**Dysfunction-discomfort**						
Total sample	1.21 (1.48)	2.50 (1.41)	**3.50 (2.79)** ^*^	4.79^▸^	**0.017**	**0.2**
Females	1.22 (1.48)	3.17 (0.75)	**4.88 (2.80)** ^*^	8.26^▸^	**0.006**	**0.43**

Values are expressed as mean and SD of the natural scores of the YFAS subscales. YFAS, Yale Food Addiction Scale; η^2^, eta squared. ^▸^ Brown-Forsythe homogeneity correction after significant test of equality of variances (Levene's). The n's for grouping participants based on their dysexecutive scores (No dysfunction, Mild-moderate dysfunction, and Severe dysfunction) for the total sample were 14, 8, 14; for females 9, 6, 8; and for males 5, 2, 6, respectively. We entered Bonferroni's *post hoc* test to allow adjustment for multiple comparisons,

* *p* < 0.05,

** *p* < 0.01 severe dysfunction vs. no dysfunction;

+ *p* < 0.05 mild-moderate dysfunction vs. severe dysfunction. Statistically significant data in bold text.

Comparisons of the specific scores of the RST-PQ subscales revealed that in the total sample, the Behavioral Inhibition System and the Punishment Inhibition System were significantly higher when the FA symptoms presence criterion was met [*t*_(34)_ = 2.93, *p* < 0.01; *t*_(34)_= 2.07, *p* < 0.05]. Specifically, females with FA had significantly higher scores on the Behavioral Inhibition and Panic Attack systems [*t*_(21)_ = 2.88, *p* < 0.01; *t*_(21)_ = 3.04, *p* < 0.001], whereas males who met the YFAS criteria had higher scores on the Punishment Inhibition and Reward Interest subscales [*t*_(11)_ = 5.16, *p* < 0.001; *t*_(11)_ = 3.49, *p* < 0.01] (Table 7). In parallel, the presence of FA was associated with significantly higher scores on the BES subscales [*t*_(34)_ = 3.55, *p* < 0.01; *t*_(34)_ = 3.15, *p* < 0.01; feelings and cognitions, behavioral manifestations, total sample], and especially in females, scores on the feelings and cognitions subscale of the BES were significantly higher [*t*_(21)_ = 3.44, *p* < 0.05]. In males, there were no significant differences in BES scores as a result of YFAS criterion fulfillment (Table 8).

**Table 7:** FA and reward sensitivity.

	**NFA (*n*= 28)**	**FA (*n*= 8)**	***t***	***p*-value**	***d***
**BIS**					
**Behavioral inhibition system**					
Total sample	30.18 (15.28)	**48.63 (17.30)** ^**^	2.93	**0.006**	1.17
Females	27.76 (17.46)	**52.00 (18.63)** ^**^	2.88	**0.009**	**1.37**
Males	33.91 (10.86)	38.50 (9.19)	0.56	0.589	0.43
**FFFS**					
**Punishment Inhibition System**					
Total sample	10.06 (7.15)	**17.50 (5.81)** ^*^	2.70	**0.011**	**1.08**
Females	12.41 (6.80)	17.83 (6.82)	1.68	0.108	0.8
Males	6.36 (6.30)	**16.50 (0.71)** ^**^	5.16	**0.001**	**1.69**
**Panic attack**					
Total sample	4.71 (3.54)	9.13 (6.06)	1.97^▸^	0.083	1.06
Females	5.00 (3.43)	**10.83 (5.56)** ^**^	3.04	**0.006**	**1.44**
Males	4.27 (3.82)	4.00 (5.66)	−0.09	0.931	0.07
**Defensive fighting**					
Total sample	10.43 (4.91)	10.88 (3.94)	0.24	0.815	0.09
Females	8.35 (4.06)	9.50 (3.33)	0.62	0.542	0.29
Males	13.64 (4.48)	15.00 (2.83)	0.41	0.692	0.31
**BAS**					
**Interest in reward**					
Total sample	11.18 (4.08)	11.00 (5.66)	−0.10	0.921	0.04
Females	10.18 (3.64)	10.33 (6.50)	0.07	0.942	0.04
Males	12.73 (4.41)	13.00 (1.41)	0.08	0.935	0.07
**Persistent Reward-seeking**					
Total sample	13.06 (3.79)	13.13 (6.10)	0.04^▸^	0.96	0.02
Females	12.88 (4.31)	12.50 (6.98)	−0.13	0.904	0.08
Males	13.27 (2.97)	15.00 (2.83)	0.76	0.463	0.58
**Reward effects**					
Total sample	17.32 (6.11)	20.88 (5.33)	1.49	0.146	0.6
Females	16.12 (6.18)	19.33 (5.32)	1.13	0.271	0.54
Males	19.18 (5.78)	**25.50 (0.71)** ^**^	3.49^▸^	**0.005**	**1.15**
**Impulsivity**					
Total sample	9.54 (4.00)	11.50 (4.38)	1.20	0.238	0.48
Females	8.94 (3.38)	10.67 (4.68)	0.97	0.341	0.46
Males	10.45 (4.82)	14.00 (2.83)	0.99	0.345	0.76

Values are expressed in terms of the mean and SD of the natural scores of the subscales of the RST-PQ. NFA, non-food addiction; FA, food addiction; BIS, Behavioral Inhibition System; FFFS, Flight, Fight, or Freeze System; BAS, Behavioral Activation System; *d*, effect size, Cohen's d. ^▸^Welch's homogeneity correction after significant test for equality of variances (Levene's). The n's for grouping participants based on the presence of FA (NFA, and FA) for the total sample were 28, 8; for females 17, 6; and for males 11, 2; respectively.

* *p* < 0.05,

** *p* < 0.01 vs. NFA. Statistically significant data in bold text.

**Table 8:** FA and binge eating.

	**NFA**	**FA**	***t***	***p*-value**	***d***
**Binge eating scale**					
**Feelings and cognitions**					
Total sample	6.18 (3.54)	**13.63 (5.63)** ^**^	3.55	**0.007**	**1.84**
Females	6.29 (3.39)	**15.00 (5.87)** ^*^	3.44	**0.013**	**2.12**
Males	6.00 (3.92)	9.5 (2.12)	1.20	0.256	0.92
**Behavioral manifestations**					
Total sample	7.86 (4.29)	**13.88 (6.24)** ^**^	3.15	**0.003**	**1.26**
Females	8.00 (4.19)	14.5 (7.00)	2.14^▸^	0.074	1.30
Males	13.27 (2.97)	15.00 (2.83)	1.22	0.250	0.94

Values are expressed in terms of the mean and SD of the natural scores of the subscales of the BES. NFA, non-food addiction; FA, food addiction; *d*, effect size, Cohen's d. ^▸^Welch's homogeneity correction after significant test for equality of variances (Levene's). The n's for grouping participants based on the presence of FA (NFA and FA) for the total sample were 28, 8; for females 17, 6; and for males 11, 2; respectively.

* *p* < 0.05,

** *p* < 0.01 vs. NFA. Statistically significant data in bold text.

### 3.4 Differences in executive dysfunction, depression, binge eating, and sensitivity to reward related to the number of FA symptoms

Using the number of FA symptoms as a grouping criterion, we found that among participants (total sample), executive dysfunction (DEX-Sp), depressive symptoms (BDI-II), and binge eating problems (BES total score and on the affective-cognitive and behavior subscales), as well as sensitivity to reward (BIS, FFFS, and BAS subscales of the RST-PQ) had significantly higher scores as more YFAS symptoms were present [*F*_(2,33)_ = 8.11; *F*_(2,33)_ = 16.10; *F*_(2,33)_ = 16.4; *F*_(2,33)_ = 12.55; *F*_(2,33)_ = 10.85; *F*_(2,33)_ = 10.52; *F*_(2,33)_ = 5.83; *F*_(2,33)_ = 15.60; *F*_(2,33)_ = 8.05; *F*_(2,33)_ = 3.42; *F*_(2,33)_ = 5.76; *p* < 0.05–0.001] (Table 9). This pattern of differences remained consistent among females who had six or more symptoms, as they had higher scores on executive dysfunction, depressive and binge eating problems (total and both subscales), as well as Fight and Freeze, Panic Attack, and Defensive Fighting from the RST-PQ [*F*_(2,20)_ = 9. 66; *F*_(2,20)_ = 14.17; *F*_(2,20)_ = 15.0; *F*_(2,20)_ = 10.53; *F*_(2,20)_ = 10.61; *F*_(2,20)_ = 9.95; *F*_(2,20)_ = 8.10; *F*_(2,20)_ = 17.11; *F*_(2,20)_ = 5.10; *p* < 0.05-0.001] (Table 10). Among males, although there were no participants with six or more symptoms of FA, those with 3–5 symptoms showed significantly greater Interest in Reward and Impulsivity (BAS of the RST-PQ) [*t*_(11)_ = 2.51, *p* < 0.05; *t*_(11)_ = 2.26, *p* < 0.05] (Supplementary Table 4).

**Table 9:** Number of FA symptoms and other cognitive and psychiatric traits, total sample.

	** ≤ 2 symptoms (*n* = 17)**	**3–5 symptoms (*n* = 15)**	**≥6 symptoms (*n =* 4)**			
	***X (SD)***	***X (SD)***	***X (SD)***	***F***	***p***	*η^2^*
**Dysexecutive symptoms**						
Natural score DEX-Sp	35.53 (7.90)	**45.80 (13.03)** ^*+^	**56.75 (9.64)** ^**^	8.11	**0.001**	**0.33**
**Binge eating**						
Natural total score BES	12.47 (6.49)	**17.2 (7.77)** ^*+++^	**35.8 (9.67)** ^***^	16.10	**0.001**	**0.49**
Feelings and cognitions	5.71 (2.93)	**7.67 (4.08)** ^**+++**^	**17.5 (5.26)** ^***^	16.40	**0.001**	**0.50**
Behavioral manifestations	6.76 (3.99)	**9.53 (4.22)** ^**++**^	**18.25 (4.5)** ^***^	12.55	**0.001**	**0.43**
**Depressive symptoms**						
Total score BDI-II	7.29 (5.41)	**16.60 (10.58)** ^*+^	**31.00 (10.89)** ^***^	10.85^▸^	**0.001**	**0.46**
**BIS**						
Natural score BIS	23.65 (12.26)	**41.27 (14.99)** ^**^	**53.25 (17.04)** ^**^	10.52	**0.001**	**0.39**
**FFFS**						
Fight-freezing	8.47 (5.35)	13.00 (7.66)	**20.50 (7.55)** ^**^	5.83	**0.010**	**0.26**
Panic attack	3.06 (2.41)	**6.73 (4.18)** ^*++^	**13.00 (3.16)** ^***^	15.60	**0.001**	**0.49**
Defensive fighting	8.06 (3.90)	**13.60 (4.03)** ^***^	9.50 (3.70)	8.05	**0.001**	**0.33**
**BAS**						
Interest in Reward	10.06 (2.99)	11.93 (4.92)	12.75 (7.14)	1.03	0.368	0.06
Reward effects	15.53 (5.84)	**20.13 (5.10)** ^*^	**21.50 (7.19)** ^*^	3.42	**0.050**	**0.17**
Impulsivity	7.82 (2.67)	**11.67 (4.50)** ^*^	12.75 (3.59)	5.76	**0.007**	**0.26**

Values are expressed as mean and SD of the natural scores of the subscales of the DEX-Sp, the BES, the RST-PQ, and the BDII. DEX-Sp, Dysexecutive Questionnaire; BES, Binge Eating Scale; BDI-II, Beck Depression Inventory-II; BIS, Behavioral Inhibition System of the RST-PQ; FFFS, Flight, Fight, or Freeze System of the RST-PQ; BAS, Behavioral Activation System of the RST-PQ; η^2^, eta squared. ^▸^Brown-Forsythe (F) homogeneity correction after a significant test for equality of variances (Levene's). We entered Bonferroni's *post hoc* test to allow adjustment for multiple comparisons, ^*^p < 0.05, ^**^p < 0.01, ^***^p < 0.001 vs. 2 or fewer YFAS symptoms; ^+^*p* < 0.05, ^++^*p* < 0.01, ^+++^*p* < 0.001 vs. 3–5 YFAS symptoms. Statistically significant data in bold text.

**Table 10:** Number of FA symptoms and other cognitive and psychiatric traits in females.

	** ≤ 2 symptoms (*n* = 12)**	**3–5 symptoms (*n* = 7)**	**≥6 symptoms (*n* = 4)**			
	***X (SD)***	***X (SD)***	***X (SD)***	***F***	***p***	*η^2^*
**Dysexecutive symptoms**						
Natural score DEX-Sp	33.92 (8.42)	42.86 (10.12)	**56.75 (9.64)** ^***^	9.66	**0.001**	**0.49**
**Binge eating**						
Natural total score BES	13.08 (7.04)	**17.14 (6.72)** ^**++**^	**35.75 (9.67)** ^**^	14.17	**0.001**	**0.59**
Feelings and cognitions	5.75 (3.17)	**8.29 (3.73)** ^**++**^	**17.50 (5.26)** ^***^	15.00	**0.001**	**0.60**
Behavioral manifestations	7.33 (4.33)	**8.86 (3.62)** ^**++**^	**18.25 (4.50)** ^***^	10.53	**0.001**	**0.51**
**Depressive symptoms**						
Total score BDI-II	7.58 (6.05)	**19.14 (12.52)** ^*^	**31.00 (10.89)** ^***^	10.61	**0.001**	**0.52**
**BIS**						
Natural score BIS	20.75 (13.50)	**46.00 (17.05)** ^**^	**53.25 (17.04)** ^**^	9.95	**0.001**	**0.50**
**FFFS**						
Fight-freezing	9.50 (4.48)	**17.43 (6.02)** ^*^	**20.50 (7.55)** ^**^	8.10	**0.003**	**0.45**
Panic attack	3.33 (2.35)	**8.29 (3.90)** ^**^	**13.00 (3.16)** ^***^	17.11	**0.001**	**0.63**
Defensive fighting	6.66 (2.93)	**11.57 (3.64)** ^*^	9.50 (3.70)	5.10	**0.016**	**0.34**
**BAS**						
Interest in Reward	10.17 (3.33)	8.86 (4.30)	12.75 (7.14)	1.00	0.372	0.09
Reward effects	14.83 (6.46)	18.00 (2.58)	21.50 (7.19)	2.20	0.136	0.09
Impulsivity	7.83 (3.13)	10.14 (3.67)	12.75 (3.59)	3.44	0.052	0.26

Values are expressed as mean and SD of the natural scores of the subscales of the DEX-Sp, the BES, the RST-PQ, and the BDII. DEX-Sp, Dysexecutive Questionnaire; BES, Binge Eating Scale; BDI-II, Beck Depression Inventory-II; BIS, Behavioral Inhibition System of the RST-PQ; FFFS, Flight, Fight, or Freeze System of the RST-PQ; BAS, Behavioral Activation System of the RST-PQ; η^2^, eta squared. We entered Bonferroni's *post hoc* test to allow adjustment for multiple comparisons, ^*^p < 0.05, ^**^p < 0.01, ^***^p < 0.001 vs. 2 or fewer YFAS symptoms. ^++^*p* < 0.01 vs. 3–5 YFAS symptoms. Statistically significant data in bold text.

### 3.5 The severity of FA and BMI is explained by dysexecutive and depressive symptoms and reward sensitivity

To assess the linear association between executive dysfunction, depressive symptoms, binge eating, food addiction, and RST-PQ scores, we calculated *rho* partial correlation coefficients while controlling for sex, BMI, and food addiction criteria. Those variables that had significant linear associations were used to feed the MLR analysis (Supplementary Figure 1).

**Figure 2:**
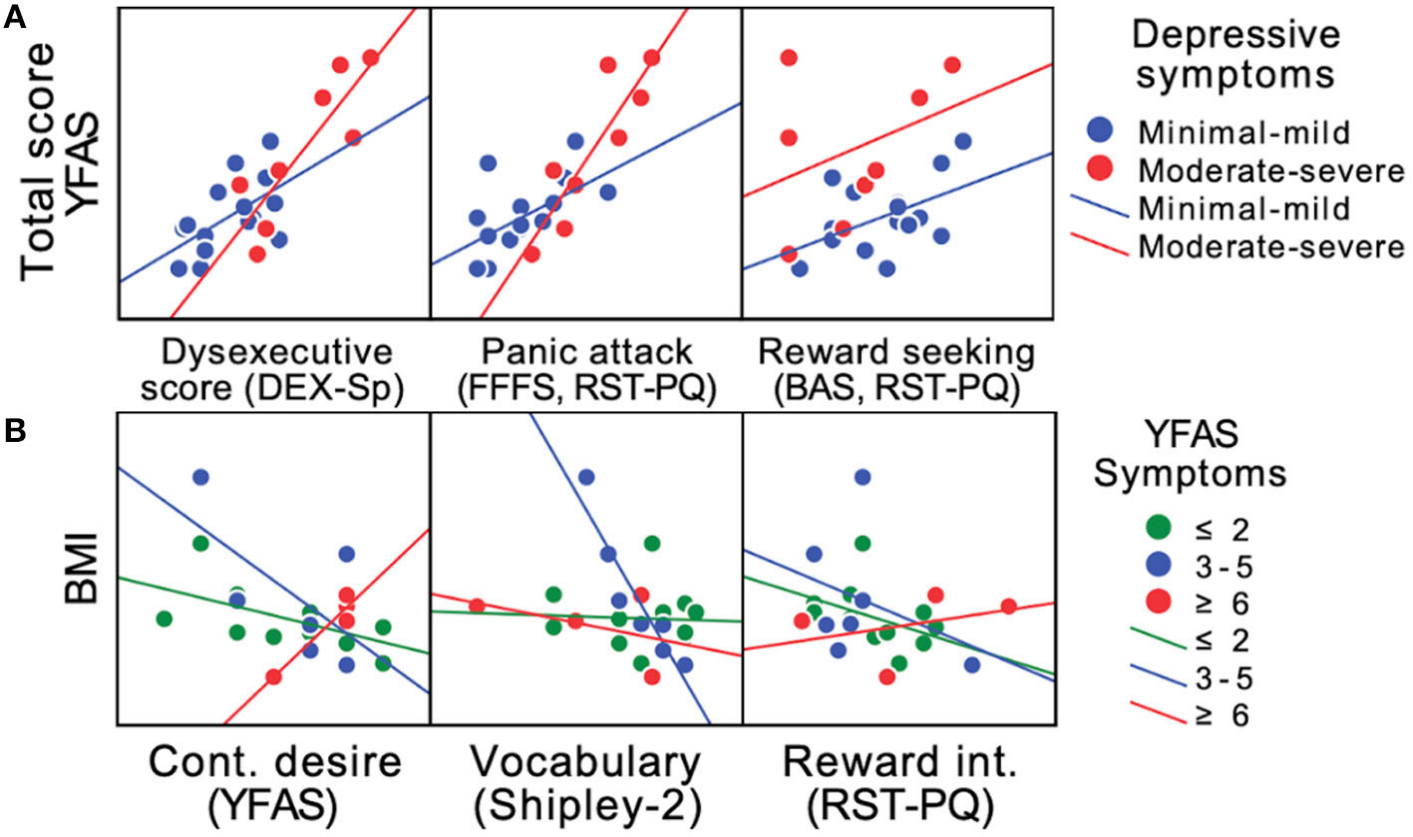
Scatterplot of changes in FA score and estimated BMI in females. Linear relationships of the main factors with significant coefficients of the RLM explaining changes in the FA score (Total score YFAS) of females in the study, considering the re-coded depressive symptoms (absent-mild, moderate-severe) **(A)**, or explaining changes in the estimated BMI of females in the study, considering the number of FA symptoms **(B)**. YFAS, Yale Food Addiction Scale; DEX-Sp, Dysexecutive Questionnaire; FFFS, RST-PQ, Fight-Flight-Freeze System (Panic Attack subscale) of the Reinforcement Sensitivity Theory of Personality Questionnaire; BAS, RST-PQ, Behavioral Approach System (Reward-seeking subscale) of the Reinforcement Sensitivity Theory of Personality Questionnaire; BMI, Body Mass Index; Cont. desire (YFAS), Continued desire subscale of the Yale Food Addiction Scale; Reward int. (RST-PQ), Reward Interest subscale of the Reinforcement Sensitivity Theory of Personality Questionnaire. Subgroup regression lines are shown [**(A)**: blue = Minimal-mild depressive symptoms; red = moderate-severe depressive symptoms; **(B)**: green = ≤ 2 symptoms; blue = 3–5 symptoms; red = ≥ 6 symptoms]. Complete results of the MLRs are presented in Supplementary Tables 5, 6.

Considering that we found linear associations between the severity of executive dysfunction, depressive symptoms, and scores on components of the Binge Eating Scale, the YFAS, and the Reward Sensitivity Questionnaire, especially in the women of the sample studied, we modeled the association between the study variables using MLR to determine whether they explained: (a) the variation in the total YFAS score (weighted by recoded depressive symptomatology, absence-mild symptoms, moderate-severe symptoms) and (b) the variation in BMI (weighted by the number of YFAS symptoms) of the women in the sample. Based on these MLR models, we found that executive dysfunction (β = 0.523, *t* = 3.523, *p* < 0.01), as well as higher scores on the RST-PQ Panic Attack (β = 0.501, *t* = 3.293, *p* < 0.01) and Reward-seeking Persistence (β = 0. 332, *t* = 3.699, *p* < 0.01) significantly explained the increase in women's total YFAS scores [*F*_(3,19)_ = 40.47; *r*${\;}_{c}^{2}$ = 0.843; *VIF* = 1.029–2.35; *p* < 0.001], especially when moderate to severe depressive symptoms were present. These results suggest that the decrease in executive function capacity, together with the activation of the Punishment Avoidance System (Panic Attack) and Persistence in Reward-seeking, is accompanied by greater intensity of FA traits as depressive symptomatology increases (Figure 2A).

Furthermore, when modeling the study variables as predictors with the MLR and using BMI as the dependent variable, we found that the subscales of continued desire of the YFAS (β = −0.374, *t* = −2.166, *p* < 0.05), vocabulary of the Shipley-2 (β = −0.729, *t* = −4.193, *p* < 0.001), as well as 3 of the subscales of the RST-PQ, Flight, Fear, and Freezing System (β = −0.6, *t* = −3.303, *p* < 0.01), Reward Interest (β = −0.506, *t* = −2.969, *p* < 0.01), and Defensive Fighting (β = 0.338, *t* = 2.131, *p* < 0.05) significantly explained the variation in the estimated females' BMI as a function of the number of FA symptoms present [*F*_(5,17)_ = 5.873; *r*${\;}_{c}^{2}$ = 0.526; *VIF* = 1.018–1.56; *p* < 0.01]. The negative coefficients suggest that the lower the crystallized intellectual capacity (Shipley 2 vocabulary), the higher the BMI, in addition to the fact that when the persistent desire or failed efforts to avoid or control food intake increase, the BMI is also higher when more FA symptoms are present (Figure 2B).

## 4 Discussion

The purpose of this study was to determine whether executive dysfunction, depressive symptomatology, binge eating problems, and high reward sensitivity entails greater severity of FA traits and elevated BMI in Mexican adults. Thus, our main finding indicated a positive correlation between the increases in executive dysfunction and scores on the YFAS scale. We also found significant correlations among depressive symptoms, YFAS subscale scores, and reward sensitivity. Although the sample included women and men, the most stable results were observed in females. Similarly, we observed that with greater impairment in decision-making ability (IGT), tolerance (YFAS) also increased, and the number of commission errors (Go/No Go) correlated positively with scores on the consumption subscale of the YFAS. An increased number of FA symptoms correlated with higher scores in executive dysfunction, greater reward sensitivity, and more severe depressive and binge eating problems, regardless of whether clinically significant impairment was present (according to the YFAS). Most importantly, we found that the factors that best explained increased scores on the AF traits were lower executive functioning in activities of daily living, accompanied by greater activation of the Punishment Avoidance System and Persistence in Reward-seeking, especially when depressive symptoms were severe. Finally, we found that the factors that best explained changes in estimated BMI were the reduced crystallized intellectual capacity and the inability to control food consumption as a function of the number of FA symptoms presented.

According to the literature, the prevalence of FA varies considerably depending on the samples studied and their clinical condition. In community samples, it has been reported that 5.4% of participants have symptoms of FA, with a ratio of approximately 2:1 between males and females (6.7 vs. 3.0%, respectively), in addition to the fact that 88.6% of those with this condition are overweight or obese (20). Meanwhile, in clinical samples, it has been reported that 24.2% may present FA (24). In the presence of an eating disorder diagnosis, the prevalence of FA may reach 57.6% (18). Accordingly, our results show that FA traits are more common in females than in males, with a ratio of 2:1.15 (26 vs. 15%, respectively, 22% in the total sample). Additionally, the presence of overweight and obesity was constant in these participants since those who met the criteria for the presence of FA had an estimated BMI >25, and especially those who had an estimated BMI >30, accumulated 87.5% of the total number of cases with FA. It is important to note that even though the sample in this study was recruited through an open call posted on social networks and could be considered a community sample, the prevalence of FA, as well as the presence of depressive symptoms, executive dysfunction, and other characteristics, strongly suggest that our participants behaved as an intentional or treatment-seeking sample (24).

Consistent with studies that have assessed executive functioning with performance tests and reported no changes attributable to the presence of FA (17, 22, 37), our observations did not show significant failures in decision-making process, cognitive flexibility, or inhibitory control assessed with the Iowa, Wisconsin, and Go/No Go tests in females with FA. We only found that when controlling for the variables of sex, BMI, and meeting the YFAS criteria for the presence of FA, deficits in decision-making and cognitive flexibility correlated with the tolerance and increased consumption subscales of the YFAS. Interestingly, after applying a questionnaire of a more ecological nature, such as the DEX-Sp, low scores on questions assessing executive function in activities of daily living were evident in females with FA. Furthermore, we found that such an executive deficit is associated with significantly high scores on four components of the YFAS, corresponding to (a) consuming more food or spending more time to obtain it, (b) withdrawal symptoms and tolerance, as well as (c) reduced social activities, and (d) dysfunction and secondary distress (25).

The discrepancy observed between the results of executive functioning measured by performance tests and self-report questionnaires may be due the fact that the first focuses on the objective assessment of cognitive functioning, while the latter allows for an ecological and subjective assessment based on the respondent's perceptions of his or her daily performance. Given this difference, a greater association between self-report questionnaires and risk behaviors has been reported in studies with adolescents, as opposed to performance tests, considering self-report measures as more consistent predictors (38), as they allow to relate the assessed executive functions to the daily context of the participants.

On the other hand, several reports in the literature indicate a high comorbidity between depression and abnormal eating behavior (21, 39, 40). In particular, a strong association between depressive symptomatology and FA severity has been reported in clinical samples, both in patients seeking or in treatment for weight control and those with eating disorders or type 2 diabetes (23, 41, 42). Our results support this association since we observed that participants in our study who were positive for the presence of FA had higher anxiety and depression scores (as assessed by the PAI) than females who did not meet the YFAS criteria. Furthermore, we found that greater severity of depressive symptoms was associated with a greater number of FA symptoms, strongly suggesting that negative emotional states may directly contribute to the intensity of FA symptoms (43), particularly in the presence of executive dysfunction and high scores on components of reward sensitivity.

The symptomatological features of binge eating and FA traits often co-occur in a significant proportion of patients with obesity and eating disorders, especially in those diagnosed with binge eating disorder, in whom the presence of FA may be present in up to 57% of cases (18). Accordingly, our results showed that the correlations between the BES scores and the components of the YFAS were positive and high, especially the feelings and cognitions subscale of BES showed significantly higher scores in females who met the criterion of the presence of at least 3 symptoms of FA and clinically significant impairment. Despite the above, we did not find a significant contribution of binge eating scores to the variation in total FA scores, which is consistent with the idea that these are two highly comorbid but clinically independent pathological entities (4, 44). In this regard, it is important to note that females who met the YFAS criterion for FA specifically had high scores on the feelings and cognitions subscale of the BES compared to females who did not meet this criterion; therefore, the eating pattern associated with FA could be abnormal, but without necessarily expressing objective binge eating.

In an attempt to characterize the abnormal eating patterns of patients with FA, it has been proposed that compulsive, repetitive, and unstructured (unplanned) consumption of small amounts of food for a significant portion of the day (grazing) is not only associated with the presence of FA but also appears to explain the severity of its symptoms (45). However, a systematic review reported that grazing is present in 33% of patients with obesity, and in patients with binge eating disorder, the prevalence can reach more than 67% (46). The above makes it difficult to differentially characterize the eating behavior pattern of patients with FA, those with binge eating disorder, and those with overweight and obesity. According to our findings, variables that characterize females with FA include poorer daily executive functioning, activation of the Punishment Avoidance System (Panic Attack), and persistence in Reward-seeking, reflecting that participants may be aware of the negative consequences of the way they consume hyper-palatable foods (thus experiencing anxiety), but are unable to delay obtaining the reward that consuming such foods provides (36), which is consistent with the idea that reward sensitivity is associated with more severe FA symptomatology (47).

Concerning psychological variables, our observations confirm and extend previous findings suggesting that FA is more common among individuals with a profile of psychological difficulties, particularly anxiety and depression (22, 41, 43, 48). In addition to displaying elevated scores on the anxiety and depression scales of the PAI, females in our sample exhibited traits indicative of borderline personality. Thus, unstable interpersonal relationships contribute to a more complex psychopathological profile when considering the presence of impaired daily executive functioning, elevated BMI, persistent desire for palatable food consumption, and impaired crystallized intellectual capacity, setting the conditions for the failure of any efforts made to control eating.

Finally, in line with other studies (49), our results also highlight the importance of the association between changes in BMI and FA symptomatology (especially continuous craving for food) and reward sensitivity (Behavioral Activation System, Interest in Reward). Specifically, participants who reported a higher BMI showed a greater number of FA symptoms and a lower ability to control food consumption, together with difficulties in problem-solving and interest in exposing themselves to situations in which they have a greater chance of obtaining a reward. Consequently, one of the main contributions of this work is the integration of cognitive characteristics from different domains that contribute to a better understanding of the factors that increase the risk of excessive weight gain.

Although this work provides new and interesting findings, it is not without limitations that should be considered. First, due to its cross-sectional design, causal relationships between the variables studied cannot be established, suggesting the need for longitudinal studies to examine whether improvements in executive functioning translate into reductions in body weight and symptoms of FA. In addition, the sample size is small, requiring future confirmatory studies with larger samples to verify the associations found between executive functioning, depressive symptoms, reward sensitivity, and FA in the Mexican adult population. Improving the advertisement procedure will be crucial to attracting more participants, especially in the case of males, considering the limited representation of this group. As a result, a cautious interpretation of the results is warranted due to the small sample size of male participants.

On the other hand, the remote application of the instruments may be subject to technical failures (e.g., instability of the internet connection). Furthermore, some of the instruments had to be adapted without standardization data in the Mexican population (IGT, Go/No-go, and WCST). The lack of more precise measurements, such as estimated BMI instead of direct measurements, also limits the results. Therefore, future studies could benefit from administering the instruments in person (e.g., including tests such as the Tower of London and Stroop in the present modality application) and obtaining more precise anthropometric measurements, such as body fat percentage, to supplement the information obtained.

In conclusion, the present study provides an exploratory characterization of the relationships among FA, executive dysfunction, depressive symptoms, and reward sensitivity in a sample of Mexican adults. To our knowledge, this is the first report to document a strong relationship between executive dysfunction and the presence of FA in Mexican women. The cognitive functioning profile, characterized by failures in general executive functioning assessed ecologically, greater activation of the Punishment Avoidance System and Persistence in Reward-seeking was associated with greater severity of FA symptoms, particularly when depressive symptomatology was severe. In parallel, the potential psychopathology present in participants with FA supports the contribution of anxious and depressive symptomatology, and borderline personality traits may contribute to the expression of clinically relevant FA symptoms. In conjunction with the above, we found that decreased crystallized intellectual capacity and inability to control food intake are associated with the presence of elevated BMI when the number of FA symptoms is higher. Finally, our results suggest that as in samples other than the Mexican sample, depressive symptoms, binge eating problems, FA, and increased reward sensitivity are conditions that often coexist. Future research should explore the observed associations in adolescents, given the increasing prevalence of overweight and obesity in younger age groups. The inclusion of face-to-face assessment instruments, the direct evaluation of anthropometric variables, and the exploration of social cognition as an understudied element of executive functioning will be critical in this regard.

## Data availability statement

The raw data supporting the conclusions of this article will be made available by the authors, without undue reservation.

## Ethics statement

The studies involving humans were approved by Ethics Committee of FES Iztacala UNAM. The studies were conducted in accordance with the local legislation and institutional requirements. The participants provided their written informed consent to participate in this study.

## Author contributions

MT-R: Conceptualization, Formal analysis, Investigation, Methodology, Writing – original draft, Writing – review & editing. AA-H: Conceptualization, Formal analysis, Funding acquisition, Methodology, Writing – original draft, Writing – review & editing. MO-L: Conceptualization, Formal analysis, Funding acquisition, Writing – original draft, Writing – review & editing. CS-J: Conceptualization, Formal analysis, Funding acquisition, Methodology, Writing – original draft, Writing – review & editing. GY-T: Conceptualization, Formal analysis, Funding acquisition, Methodology, Writing – original draft, Writing – review & editing. VL-A: Conceptualization, Formal analysis, Funding acquisition, Writing – original draft, Writing – review & editing. JM-D: Formal analysis, Funding acquisition, Methodology, Project administration, Writing – original draft, Writing – review & editing. RE-P: Conceptualization, Formal analysis, Funding acquisition, Methodology, Project administration, Resources, Supervision, Writing – original draft.
