# Supplementary material for: Exploratory study of the prevalence of food addiction and its relationship with executive functioning, depression, and reinforcement sensitivity in a sample of Mexican adults
Source: Front Public Health. 2023 Dec 1;11:1277681. doi: 10.3389/fpubh.2023.1277681 (PMC10722417; doi:10.3389/fpubh.2023.1277681)
Supplement: Supplementary file 1 [file Table_1.pdf]

Supplementary Table 1. FA, sex, BMI, and neuropsychological evaluations in males

	NFA (*n*= 11)	FA (*n*= 2)	*t*	*p*-value	*d*
**Measures**					
BMI ○	28.01 (5.69)	32.06 (0.77)	0.97	0.353	1.00
Weight ○	83.12 (16.97)	95.50 (7.78)	0.99	0.346	0.94
Height ○	1.72 (0.05)	1.73 (0.05)	0.08	0.937	0.20
Age	27.91 (5.38)	33.00 (9.90)	1.12	0.288	0.64
**Intellectual ability**					
Vocabulary Shipley-2 ▽	108.91 (3.27)	111.5 (4.95)	0.98	0.350	0.62
Abstraction Shipley-2 ▽	101.27 (10.00)	105.00 (4.95)	0.51	0.621	0.47
Total ▽	111.36 (7.45)	116.36 (2.12)	0.94	0.369	0.91
**Decision-making (IGT)**					
Total net score	16.18 (34.54)	30.00 (0.01)	0.55	0.596	0.57
**Cognitive flexibility (WCST)**					
Perseverative errors (%)	19.55 (9.87)	**8.00 (0.01)****	-3.88	**0.003**	**1.65**
Commission errors	4.27 (4.69)	1.00 (1.41)	-0.95	0.364	0.94
**Psychopathology (PAI)**					
Drug problems □	51.64 (9.78)	**44.00 (0.01)***	-2.59	**0.027**	**1.10**
Alcohol problems □	52.45 (14.12)	44.00 (0.01)	-0.82	0.431	0.85
**Dysexecutive symptoms (DEX-Sp)**					
Dysexecutive score	45.82 (13.39)	40.00 (12.73)	-0.57	0.581	0.45
**Binge eating (BES)**					
Total score	13.64 (8.14)	21.5 (6.36)	1.28	0.227	1.08
**Depressive symptoms (BDI-II)**					
Total score	11.82 (8.70)	9.00 (4.24)	-0.44	0.671	0.41
**Reward sensitivity (RTS-PQ)**					
Total score	113.82 (27.09)	141.5 (21.92)	1.35	0.204	1.11

Values are expressed as means and their SDs. Standard scores have a mean of 100 and a SD of 15, while t scores have a mean of 50 and a SD of 10. ○ Estimates based on questionnaire responses; ▽ Standard score; □ t score; d = effect size, Cohen's d. NFA= non-food addiction; FA= food addiction; BMI= body mass index; IGT= Iowa Gambling Test; PAI= Personality Assessment Inventory; DEX-Sp= Dysexecutive Questionnaire; BES= Binge Eating Scale; BDI-II= Beck Depression Inventory-II; RST-PQ= Reinforcement Sensitivity Theory of Personality Questionnaire. *$p< 0.05$, **$p< 0.01$ versus NFA.

Supplementary Table 2. YFAS subscales and BMI

	**BMI<25**	**25≤BMI<30**	**BMI≥30**			
**Food addiction (YFAS)**	***X (SD)***	***X (SD)***	***X (SD)***	***F***	***p-value***	η^2
**Increased consumption**						
Total sample	17.00 (8.46)	13.62 (5.10)	18.67 (9.60)	1.68	0.202	0.09
Females	22.00 (6.56)	12.50 (5.93)	18.17 (10.46)	1.64	0.220	0.14
Males	9.50 (3.54)	14.75 (4.20)	20.67 (6.03)	3.74	0.061	0.43
**Tolerance**						
Total sample	2.80 (2.95)	3.13 (2.73)	5.07 (3.24)	2.03	0.148	0.11
Females	4.67 (2.08)	4.13 (3.40)	4.92 (3.60)	0.13	0.879	0.01
Males	0.0 (0.0)	2.13 (1.46)	5.67 (2.26)	-	-	-
**Continuous craving**						
Total sample	6.60 (1.52)	5.75 (1.24)	5.13 (1.81)	1.83	0.117	0.10
Females	6.67 (1.53)	6.13 (1.25)	5.08 (1.93)	1.55	0.326	0.13
Males	6.5 (2.12)	5.38 (1.19)	5.33 (1.53)	0.58	0.58	0.01
**Reduced social activities**						
Total sample	1.60 (1.52)	1.81 (1.76)	3.13 (3.14)	1.42	0.256	0.08
Females	2.67 (0.58)	2.38 (2.20)	3.17 (3.54)	0.18	0.837	0.02
Males	0.0 (0.0)	1.25 (1.04)	3.00 (0.0)	-	-	-
**Dysfunction-discomfort**						
Total sample	1.6 (1.52)	1.75 (1.88)	3.33 (2.61)	2.41	0.105	0.13
Females	2.67 (0.58)	2.50 (2.33)	3.42 (2.84)	0.35	0.712	0.03
Males	0.0 (0.0)	1.0 (0.93)	3.00 (1.73)	-	-	-

Values expressed as mean and SD of the natural scores of the YFAS subscales. BMI= Body Mass Index; YFAS= Yale Food Addiction Scale; η^2= eta squared; - not calculated. The n's for grouping participants based on their BMI (<25, 25≤BMI<30, and BMI≥30) for the total sample were 5, 16, 15; for females, 3, 8, 12; and for males, 2, 8, 2, respectively. We entered Bonferroni's Post hoc test to allow adjustment for multiple comparisons, **p*< 0.05, ***p*< 0.01 vs BMI<25.

Supplementary Table 3. YFAS subscales and dysexecutive symptoms (DEX-Sp) in males

	No dysfunction	Mild-moderate dysfunction	Severe dysfunction			
**Food addiction (YFAS)**	***X (SD)***	***X (SD)***	***X (SD)***	***F***	***p*-value**	η^2
**Increased consumption**						
Males	15.40 (7.09)	10.50 (4.95)	16.83 (3.97)	0.99	0.406	0.17
**Tolerance**						
Males	2.20 (2.86)	0.50 (0.71)	3.67 (1.51)	1.84	0.208	0.27
**Continuous craving**						
Males	6.60 (1.14)	4.50 (0.71)	5.00 (1.10)	4.07	0.051	0.45
**Reduced social activities**						
Males	0.80 (1.30)	0.50 (0.71)	2.33 (0.82)	4.04	0.052	0.45
**Dysfunction-discomfort**						
Males	1.20 (1.64)	0.50 (0.71)	1.67 (1.51)	0.47	0.637	0.09

Values are expressed as mean and SD of the natural scores of the YFAS subscales. YFAS= Yale Food Addiction Scale; η^2= eta squared. ‣ Brown-Forsythe homogeneity correction after significant test of equality of variances (Levene's). The n's for grouping participants based on their dysexecutive scores (No dysfunction, Mild-moderate dysfunction, and Severe dysfunction) for the total sample were 14, 8, 14; for females 9, 6, 8; and for males 5, 2, 6, respectively. We entered Bonferroni's Post hoc test to allow adjustment for multiple comparisons, *$p< 0.05$, **$p< 0.01$ severe dysfunction vs no dysfunction; $^+p< 0.05$ mild-moderate dysfunction vs severe dysfunction.

Supplementary Table 4. Number of FA symptoms and other cognitive and psychiatric traits in males

	**≤ 2 symptoms (*n* = 5)**	**3-5 symptoms (*n* = 8)**			
	X (SD)	***X (SD)***	***t***	***p-value***	***d***
**Dysexecutive symptoms**					
Natural score DEX-Sp	39.40 (5.32)	48.38 (15.35)	2.29‣	0.16	0.12
**Binge eating**					
Natural total score BES	11.00 (15.34)	17.25 (9.05)	1.39	0.193	0.15
Feelings and cognitions	5.60 (2.60)	7.12 (4.55)	0.67	0.513	0,04
Behavioral manifestations	5.40 (2.97)	10.13 (4.83)	1.94	0.078	0.26
**Depressive symptoms**					
Total score BDII	6.60 (3.91)	14.38 (8.80)	1.84	0.093	0.24
**BIS**					
Natural score BIS	30.60 (3.85)	37.13 (12.59)	1.11	0.29	0.10
**FFFS**					
Fight-freezing	6.00 (6.96)	9.13 (7.04)	0.78	0.451	0.05
Panic Attack	2.40 (2.70)	5.38 (4.17)	1.41	0.187	0.15
Defensive fighting	11.40 (4.16)	15.38 (3.66)	1.81	0.098	0.23
**BAS**					
Interest in Reward	7.80 (1.30)	**13.00 (4.96)***	2.51	**0.029**	**0.37**
Reward effects	17.20 (4.09)	22.00 (6.14)	1.54	0.153	0.18
Impulsivity	7.80 (1.30)	**13.00 (4.96)***	2.26	**0.045**	**0.32**

Values are expressed as mean and SD of the natural scores of the subscales of the DEX-Sp, the BES, the RST-PQ, and the BDII. DEX-Sp= Dysexecutive Questionnaire; BES= Binge Eating Scale; BDII= Beck Depression Inventory-II; BIS= Behavioral Inhibition System of the RST-PQ; FFFS= Flight, Fight, or Freeze System of the RST-PQ; BAS= Behavioral Activation System of the RST-PQ; *d*= effect size, Cohen's d. ‣ Welch's (t) homogeneity correction after a significant test for equality of variances (Levene's). *$p < 0.05$ versus 2 or fewer YFAS symptoms.

Supplementary Table 5. Results from MLR YFASTotal

**Model summary – YFASTotal**									
Model	*R*	*R^2*	*Adjusted R^2*	*RMSE*	*R^2 Change*	*F Change*	*df1*	*Df2*	*p*
H_0	0.000	0.000	0.000	20.925	0.000		0	22	
H_1	0.930	0.865	0.843	8.283	0.865	40.470	3	19	< 0.001

**ANOVA**						
Model		*Sum of squares*	*df*	*Mean square*	*F*	*p*
H_1	Regression	8329.223	3	2276.408	40.470	< 0.001
	Residual	1303.478	19	68.605		
	Total	9632.710	22			

**Coefficients**								
							Collinearity Statistics	
Model		*Unstandardized*	*Standard Error*	*Standardized*	*t*	*p*	*Tolerance*	*VIF*
H_0	(Intercept)	34.903	3.758		9.287	< 0.001		
H_1	(Intercept)	- 22.453	7.445		- 3.016	0.007		
	ExecDysfunction	0.705	0.200	0.523	3.523	0.002	0.431	2.320
	PanAttackRST-PQ	1.741	0.529	0.501	3.293	0.004	0.424	2.359
	RewSeekPRST-PQ	1.100	0.297	0.332	3.699	0.002	0.971	1.029

Supplementary Table 6. Results from MLR BMI

**Model summary – BMI**									
Model	*R*	R^2	*Adjusted* R^2	*RMSE*	R^2 *Change*	*F Change*	*df1*	*Df2*	*p*
H_0	0.000	0.000	0.000	12.059	0.000		0	22	
H_1	0.796	0.633	0.526	8.306	0.633	5.873	5	17	0.002

**ANOVA**						
Model		*Sum of squares*	*df*	*Mean square*	*F*	*p*
H_1	Regression	2026.189	5	405.238	5.873	0.002
	Residual	1172.962	17	68.998		
	Total	3199.151	22			

**Coefficients**								
							Collinearity Statistics	
Model		*Unstandardized*	*Standard Error*	*Standardized*	*t*	*p*	*Tolerance*	*VIF*
H_0	(Intercept)	31.274	1.672		18.702	< 0.001		
H_1	(Intercept)	181.458	32.458		5.535	< 0.001		
	ContDesireYFAS	-1.718	0.793	- 0.374	- 2.166	0.045	0.847	1.181
	VocShipley2	-1.162	0.277	- 0.729	- 4.193	< 0.001	0.763	1.311
	FFFSRST-PQ	- 0.669	0.203	- 0.600	- 3.303	0.004	0.642	1.558
	RewIntRST-PQ	- 0.911	0.307	- 0.506	- 2.969	0.009	0.658	1.521
	DefFightRST-PQ	0.694	0.3.26	0.338	2.131	0.048	0.768	1.302

Supplementary Figure 1. Partial correlations (*rho*), control variables: Sex, Obesity, FA presence

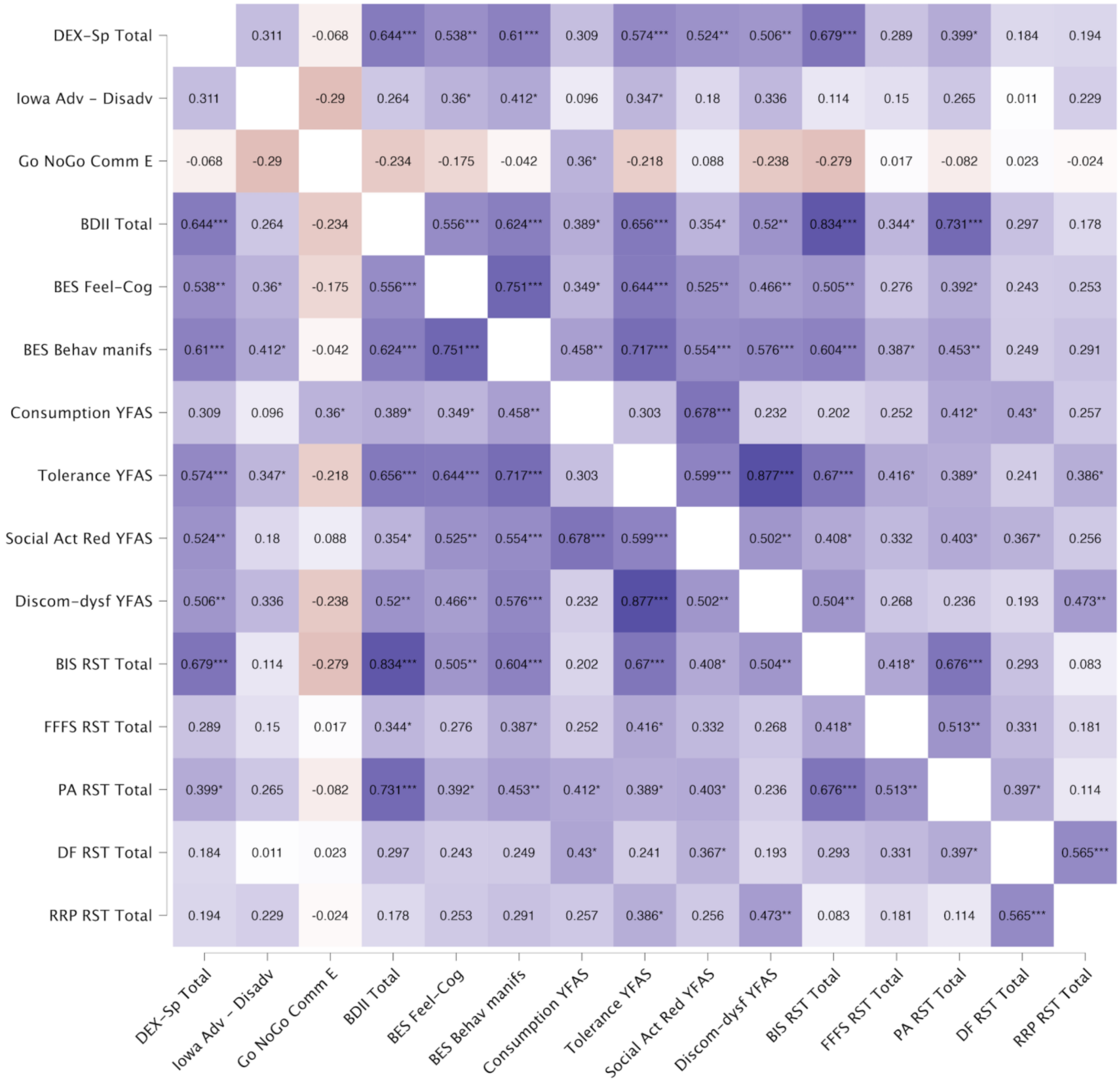

Partial correlations (rho) controlled for sex, BMI > 25, and meeting FA criteria, between scores on tests assessing executive functioning (DEX-Sp, Iowa, Go-No Go), depressive symptoms (BDI-II), binge eating symptoms (BES), FA (YFAS), and reward sensitivity (RST-PQ). DEX-Sp= Total score Dysexecutive Questionnaire, Iowa Adv-Disadv= Iowa Gambling Test total score, Go NoGo Comm E= Go/ No-go commission errors, BDII Total= Beck Depression Inventory-II total score, BES Feel-

Cog= Binge Eating Scale feelings and cognitions subscale total score, BES Behav Manifs= Binge Eating Scale Behavioral Manifestations subscale total score, Consumption YFAS= Yale Food Addiction Scale Consumption dimension score, Tolerance YFAS= Yale Food Addiction Scale Tolerance subscale score, continued use despite knowledge of negative consequences, Social Act Red YFAS= Yale Food Addiction Scale Reduction or cessation of social occupational or recreational activities subscale total, Discomf-Dysf YFAS= Yale Food Addiction Scale Consequences of discomfort and dysfunction due to reduction or cessation of social occupational or recreational activities subscale total, BIS RST Tot= Reward Sensitivity Theory-PQ Behavioral Inhibition System subscale total, FFFS RST Total= Reward Sensitivity Theory-PQ Flight, Fight or Freeze System subscale total, PA RST Total= Reward Sensitivity Theory-PQ Panic Attack subscale total, DF RST Total = Reward Sensitivity Theory-PQ Defensive Fighting subscale total, RRP RST Total= Reward Sensitivity Theory-PQ Reward Effects subscale total, $n = 36$. *$p < 0.05$, **$p < 0.01$, ***$p < 0.001$.
